# Development and testing of a mobile application to support diabetes self-management for people with newly diagnosed type 2 diabetes: a design thinking case study

**DOI:** 10.1186/s12911-017-0493-6

**Published:** 2017-06-26

**Authors:** Mira Petersen, Nana F. Hempler

**Affiliations:** Health Promotion Research, Steno Diabetes Center Copenhagen, Niels Steensens Vej 6, Copenhagen, 2820 Gentofte Denmark

**Keywords:** Mobile application, Type 2 diabetes, Diabetes support, Diabetes self-management, Design thinking, Qualitative methods

## Abstract

**Background:**

Numerous mobile applications have been developed to support diabetes-self-management. However, the majority of these applications lack a theoretical foundation and the involvement of people with diabetes during development. The aim of this study was to develop and test a mobile application (app) supporting diabetes self-management among people with newly diagnosed type 2 diabetes using design thinking.

**Methods:**

The app was developed and tested in 2015 using a design-based research approach involving target users (individuals newly diagnosed with type 2 diabetes), research scientists, healthcare professionals, designers, and app developers. The research approach comprised three major phases: inspiration, ideation, and implementation. The first phase included observations of diabetes education and 12 in-depth interviews with users regarding challenges and needs related to living with diabetes. The ideation phrase consisted of four interactive workshops with users focusing on app needs, in which ideas were developed and prioritized. Finally, 14 users tested the app over 4 weeks; they were interviewed about usability and perceptions about the app as a support tool.

**Results:**

A multifunctional app was useful for people with newly diagnosed type 2 diabetes. The final app comprised five major functions: overview of diabetes activities after diagnosis, recording of health data, reflection games and goal setting, knowledge games and recording of psychological data such as sleep, fatigue, and well-being. Users found the app to be a valuable tool for support, particularly for raising their awareness about their psychological health and for informing and guiding them through the healthcare system after diagnosis.

**Conclusions:**

The design thinking processes used in the development and implementation of the mobile health app were crucial to creating value for users. More attention should be paid to the training of professionals who introduce health apps.

Trial registration: Danish Data Protection Agency: 2012-58-0004. Registered 6 February 2016.

## Background

In Denmark and internationally, the prevalence of type 2 diabetes is growing rapidly, especially in high-income countries [1]. The increase is related to aging populations, economic development, a less healthy diet, increasing urbanization, and reduced physical activity [1]. As a consequence, diabetes has a significant economic impact on nations and national health systems because of increased use of health services, lower productivity, and the need for long-term support to reduce diabetes-related complications [2, 3].

Consequently, in diabetes care, self-management and education are considered core elements of reducing risk factors and long-term disability and preventing diabetes-related complications [4, 5]. People with type 2 diabetes provide the majority of their own care between clinic visits that total less than two hours of formal diabetes care per year [6]. However, advances in smartphone technology have led to new opportunities for supporting diabetes self-management and delivering diabetes education. A promising approach is Mobile Health (mHealth). MHealth is defined as “medical and public health practices supported by mobile devices, such as mobile phones, patient monitoring devices, personal digital assistants and other wireless devices [7]. Benefits related to diabetes care may include improved health behavior and clinical outcomes, an easier transition to life with diabetes, and increased access to the healthcare system brand [8–11].

The adoption of smartphones by the general public has increased dramatically. In Denmark, 77% of all families own at least one smartphone and 50% own a tablet [12]. The rising numbers emphasize the potential for developing mobile applications (apps) as support tools for diabetes self-management. A multitude of diabetes apps are already available. As shown by Vitger et al., the number of diabetes apps available through Apple’s App Store has increased steadily from approximately 600 in 2003 to more than 1000 by 2015 [13].

Although the field of mHealth is still in its infancy, some studies have explored mobile apps in relation to diabetes self-management [8–10, 13, 14]. The general findings show that the majority of the apps studied lacked a theoretical foundation and did not involve the needs and preferences of the target group in the development process. Some ‘diabetes management’ apps do not follow medical guidelines or incorporate clinical best practices established by diabetes professionals [8]. Several apps have usability issues, and app functions focus narrowly on insulin dosage suggestions, recording medications, and diet and weight management. Several studies identify a need to employ a more user-centered and holistic approach in which the target group is actively involved in developing and testing the usability and usefulness of an app [10, 13, 14].

The objective of this study was to use the principles of design thinking to collaborate with newly diagnosed individuals with type 2 diabetes to create an app supporting diabetes self-management and test the usability and usefulness of the app.

## Methods

The study was conducted collaboratively by the Capital Region of Denmark, the Municipality of Copenhagen, an information technology company, a general practice, and Steno Diabetes Center. Collaborators shared roles and responsibilities, such as recruiting patients, validating content of the app in relation to evidence-based clinical guidelines, and app design and development. The study was conducted on the basis of a public grant for the development of digital solutions for management of newly diagnosed type 2 diabetes.

Individuals who were newly diagnosed with type 2 diabetes were involved in all processes. Each activity such as workshops, observations, interviews or testing involved new participants (users); only one user participated in all three phases of the study. In addition, we involved healthcare professionals (five GPs, a physician, two diabetes nurses and two GP secretaries) in app development and testing. Researchers from Steno Diabetes Center with backgrounds in public health, behavioral, and educational science were the primary investigators of the study, which took place between December 2014 and January 2016.

### Study design

We applied the methodology of design thinking, which is an innovative human-centered approach to developing new solutions [15]. Design thinking addresses the needs of the people who will consume a product and the infrastructure that enables it [15]; it takes into account the perspectives of multiple stakeholders. In this case, patients newly diagnosed with type 2 diabetes were the primary users of the app (referred to hereafter as ‘users’), but healthcare professionals were also stakeholders and were involved closely in the design process.

Design thinking focuses on rapid prototyping, which means turning ideas into actual products that are then tested, iterated, and refined, based on user feedback [15]. The study process was inspired by Brown and Wyatt’s three phases of inspiration, ideation, and implementation [15], which are depicted in Table 1. In practice, the phases overlap and iterate. We used qualitative methods such as observations, semi-structured interviews, and interactive workshops to promote participation with users and healthcare professionals in developing and testing the app. Tools and methods from the educational concept ‘Next Education’ (NEED) were applied to promote active involvement, reflection, and dialog in workshops [16–18].

**Table 1:** Types of data generated in workshops, observations and individual interviews using a design thinking approach

Three phases		Aim	Participants (n)	Methods	Data
Phase 1 - inspiration	Workshop 1 (HCP^a^)	To identify needs and challenges in the communication between users and HCP	9	Dialogue tools	Minutes Video recordings
	Observations (Individual consultation in GP, patient education in CHC	To identify user needs and challenges To gain insight into the working procedure and consultation/ patient education To recruit users for a workshop	11	Open observation strategy	Field notes
	Individual Interviews	To identify user needs and challenges	12	Semi-structured individual interviews	Transcribed individual interviews
	Workshop 2 (Users)	To validate the results from workshop 8 1, the individual interviews and the observations To identify and discuss users’ needs and challenges	8	Personas of user needs and challenges, Visualized ideas	Audio-recorded Minutes
Phase 2- ideation	Workshop 3 (Users)	To discuss five refined ideas To refine and adjust the ideas	8	Visualized ideas, User Journey, Flowchart	Audio- recorded Minutes
	Workshop 4 (Users)	To discuss seven refined ideas To prioritize the ideas with the participants To refine and adjust the ideas	7	Visualized ideas, Dialogue tools	Audio- recorded Minutes
	Individual Interview	To discuss seven refined ideas To prioritize the ideas To refine and adjust the ideas Minutes with users	1	Visualized ideas, Dialogue tools	Audio-recorded Minutes
	Workshop 5 (Users)	To get feedback on the preliminary app content and design To refine and adjust the wireframes	9	Wireframes	Minutes
	Individual Interviews (GPs)	To get feedback on the preliminary app content and design To discuss a potential implementation of the app in practice	3	Wireframes	Minutes
Phase 3 - implementation	Observations (GP, CHC)	To observe introductions to the app conducted by HCP		Open observation strategy	Minutes
	Individual interviews	To explore users’ experiences with the prototype in practice	14	Semi-structured individual interview	Transcribed individual interviews
	Workshop 6 (HCP^a^)	To discuss a potential implementation of the app in practice based on the pilot study and the HCP’s experiences	7	Visualized recruitment overview of app in practice	Minutes

^a^HCP workshops included GPs, a physician (only workshop 1), two GP secretaries (only workshop 6) and two diabetes nurses

Abbreviations: *GP* general practitioner, *HCP* healthcare professional, *CHC* Community health center

### Phase 1: inspiration

The inspiration phase focused on understanding users’ needs and challenges in everyday life after diagnosis with type 2 diabetes. Users were recruited in a general practice and a community health center providing patient education in the municipality of Copenhagen. We conducted an interactive workshop with healthcare professionals (e.g., general practitioners [GPs] and diabetes nurse specialists), observations of patient education in a community health center and individual consultations in general practice as well as 12 semi-structured individual interviews with users. We intended to include individuals who had been diagnosed within the previous 2 years, but we broadened this criterion to facilitate recruitment; users’ duration of disease ranged up to 10 years. We asked users who had been diagnosed longer than 2 years to focus on their needs and challenges in the period after diagnosis.

Data collection focused on three themes: everyday life with type 2 diabetes, communication with the healthcare system, and technology knowledge and readiness. An interview guide was compiled, based on recommendations from Brinkmann and Tanggaard [19]. The guide also included questions related to demographics, such as age, education, employment status, and marital status, and questions related to diabetes, such as use of medication, blood sugar monitoring and control, disabilities, and other chronic diseases (Table 2). The semi-structured interviews were inspired by the model ‘The Balancing Person’, which describes patients’ challenges with a chronic condition [20]. The interviews varied in length from 20 to 60 min and were transcribed verbatim; the findings were validated in a workshop with users.

**Table 2:** Participant characteristics – individual interviews (phase one)

Female (*n*)	7
Male (*n*)	5
Age, mean, (range), years	56 (43-70)
Diabetes duration, mean, (ranges), years	2.5 (0-11)
Employed (*n*)	7
Retired (*n*)	5
Married/living with a partner (*n*)	6
Own a smartphone/tablet (*n*)	10

### Phase 2: ideation

In the ideation phase, we analyzed data and ransformed them into insights about innovative solutions for change [15]. Analysis was inspired by Malterud’s ‘systematic text condensation’ [21]. First, we captured an overall impression of all data and then identified preliminary themes. Secondly, we identified the meaning units relevant to the study question and sorted them into categories representing different themes. Thirdly, the units of meaning were sorted into subgroups and the meaning in each subgroup was refined and condensed. Finally, the content of the subgroups was synthesized to generate descriptions and concepts [21].

We conducted three interactive workshops with users and healthcare professionals. The purpose was to develop, discuss, and prioritize ideas for app content and design. We used tools from the Next Education concept, visualized ideas, and used dialog tools such as a flowchart of a user journey experience inspired by participants’ experiences and challenges. The number of ideas, app content, and design were adjusted and refined throughout the ideation process. Relevant app content was validated by the study collaborators in general practice, the community health center, the hospital and by the Danish Diabetes Association, the Danish Podiatry Association as well as the Eye Clinic at Steno Diabetes Center in Copenhagen.

### Phase 3: implementation

The app prototype was pilot tested with users for a period of 4 weeks. Fourteen users were recruited from general practice and the community health center (Table 3). Further three users had agreed to test the app, but dropped out of the study before they had downloaded the app due to family circumstances, lack of acceptance and readiness in relation to a diabetes diagnosis, and technical issues. An inclusion criterion was access to an iPhone or iPad because the app was only developed for Apple’s iOS platform. Users agreed to test the app for 4 weeks and participate in an interview regarding usability and usefulness. To assist healthcare professionals in the recruitment process, we created both a script and an information letter to give to users who showed interest in the pilot study.

**Table 3:** Participant characteristics – app test (phase three)

Female (*n*)	7
Male (*n*)	7
Age, mean, (range), years	52 (33-64)
Diabetes duration, mean (range), years	3 (0-16)
Employment (*n*)	8
Married/living with a partner (*n*)	10

To access the app, users installed a secure development platform app that required an invitation with a username and a password. We observed the processes of recruitment and introduction to the app to gain insight into potential implementation challenges. For this purpose, we developed an observation guide that contained questions related to how recruitment was conducted in practice, the types of questions that potential users had, and the characteristics of people who declined to participate.

We conducted semi-structured interviews with 14 users. They focused on the participant’s experience with the app and also included data such as duration of disease, age, education, employment status, and marital status (Table 3). The interviews, which lasted 22 to 55 min, were transcribed verbatim. Furthermore, implementation issues were discussed in a workshop with healthcare professionals. The findings from interviews and the workshop were used to create a list of recommended adjustments to the app.

## Results

In the process of developing and testing the app, we conducted 6 workshops and 26 interviews with people with newly diagnosed type 2 diabetes.

### Themes identified in the inspiration phase

The analysis of data interviews, observations, and workshops from the needs assessment in Phase 1 revealed four themes: 1) diabetes – a real illness?; 2) lack of action competency; 3) non-transparent diabetes journey; and 4) lack of care coordination.

#### Diabetes - a real illness?

Most users were symptom free and stated that they had no complications of diabetes. Several users considered diabetes to be an illness from which one could suffer to varying degrees, and they identified themselves as being on the low end of that continuum. The husband of one user described her condition as ‘diabetes light’. The lack of perceived illness had a negative impact on users’ diabetes self-management, which affected their motivation to participate in diabetes education.

#### Lack of action competency

Users described receiving recommendations to ‘eat healthy’, ‘stop eating sweets’, and ‘lose weight’ from healthcare professionals after diagnosis. However, they reported a need for concrete, simple information about diabetes and about how to integrate changes into their daily lives. Several users also reported a gap between the type of information they received and the type of information they needed.

#### Non-transparent diabetes journey

Users reported a lack of overview of diabetes care activities, such as visits to podiatrists and eye specialists, patient education in community health centers, the Danish Diabetes Association, and the like. Nevertheless, they were very interested in being introduced to these activities by their GP. Several users mentioned they were disappointed that they had not been informed about or referred to particular activities after diagnosis.

#### Lack of care coordination

**Fig. 1:**
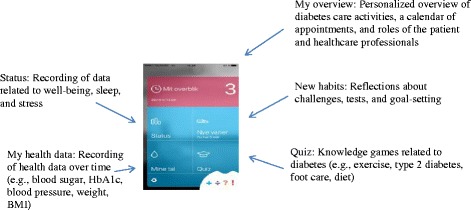
Summary of feedback from workshops with users

Users often described the period of time following a diagnosis of type 2 diabetes as difficult because they found it hard to navigate the healthcare system (Fig. 1). They suddenly needed to coordinate a great deal of information and keep track of numerous appointments with different healthcare professionals. Users often described this experience as extremely stressful and time-consuming. In addition, some users felt lonely and insecure in their role as ‘coordinator of information’. Some users also received conflicting information from different healthcare professionals about how to manage their diabetes.

### Solutions identified in the ideation phase

**Fig. 2:**
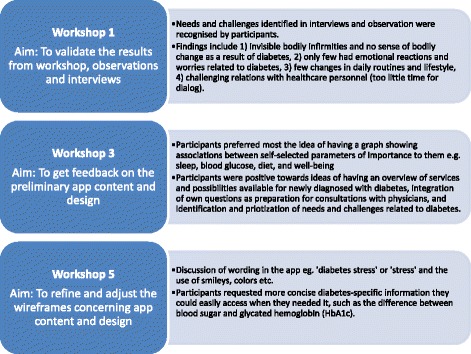
App functions

Gaining a deep understanding of users’ needs inspired two solutions for an app prototype. The first solution aimed to strengthen users’ ability to navigate diabetes care activities and coordinate information (health data) by providing an overview of vital diabetes care activities and stimulating reflection about their diabetes-related needs, goals, and challenges. The second solution focused on supporting newly diagnosed individuals in making health behavior changes and maintaining psychosocial health by addressing well-being, stress, and sleep. During discussions about the relative priority of these solutions with users and healthcare professionals, a collaborative decision was reached to integrate both solutions into a single app prototype (Fig. 2). After prototyping was completed, the app comprised five functions:

1. overview of diabetes activities after diagnosis
  This function includes an overview of the resources in the local community in relation to diabetes care. Resources are listed by options and activities e.g. health activities in the community health center, podiatrist and GPs. It is possible to add appointments for each activity and personalize the overview. There is also short information associated with the activities such as ‘you can prevent complications by…’ and ‘why see a podiatrist?’ etc.
2. recording of and knowledge about health data
  The function includes short and concise knowledge of blood sugar, including old and new measurement, HbA1c, blood pressure, cholesterol, weight and BMI. The user can add values, set goals and watch his or her ‘health data history’.
3. a reflection game about challenges and goal setting,
  This function of the app makes it possible to set goals within the categories “My disease and I”, “Food” and “Exercise”. The user can also test if he or she shares the same challenges as other patients with type 2 diabetes (by prioritizing real patient quotes). The aim of this function is to promote reflection of priorities and challenges of living with diabetes.
4. knowledge games
  This function contains 4 quizzes; “Exercise”, “Type 2 diabetes”, “Feed”, and “Food and diabetes”. After each quiz the user may find information and links about options and activities relating to the subject of the quiz.
5. recording of psychosocial data, such as sleep, fatigue, and well-being
  This function of the app contains psychosocial data which the user can assess each day (using smileys). It is possible to visualize in graphs. The data contain the questions: 1 “Have you been feeling nervous and stressed?”, 2 “Do you feel that you get enough sleep and do you feel rested?” and 3 “How are you doing?”.

### Themes identified in the implementation phase

We identified four themes from the interviews with users and from a focus group with healthcare professionals about implementation. The themes were: 1) a viable tool to support diabetes self-management, 2) patterns of app use, 3) barriers and facilitators of app use, and 4) barriers and facilitators of implementation.

#### A viable tool to support diabetes self-management

The findings suggested that the app was a viable tool to support diabetes self-management among people with type 2 diabetes. It provided assistance in initiating or maintaining lifestyle changes, routines, or habits in daily life. Many users stated they would continue using the app if it was optimized technically and some app features were adjusted, such as receiving continuous feedback in knowledge games in addition to a final score (Table 4). Several users reported that the app provided a useful overview of diabetes-related activities, which improved their ability to navigate both the healthcare system and local diabetes activities. Information on diabetes was very useful, particularly information about preventive diabetes activities that included different roles and responsibilities. Users also reported that the app contained concise diabetes-specific information they could easily access when they needed it, such as the difference between blood sugar and glycated hemoglobin (HbA1c). Furthermore, users described the self-reported data about sleep, stress, and well-being as promoting awareness about how they could improve these areas of their lives. Some users reported that increasing awareness of their own health and well-being from the app improved their decision-making about their health. One user (male, 64 years old) said that recording his weight in the app had motivated him to eat a healthier diet. Another participant (female, 46 years old) gained important insight into her daily routines by using the self-reported outcomes (e.g., sleep, stress, and well-being) visualized in graphs, which led her to make changes in her daily routines.

**Table 4:** Self-reported app use

	n
Device downloads	
iPhone	9
iPad	5
App use over 4 weeks	
Less than 3 times in total	2
Once a week	1
Several times a week	9
On a daily basis	2
Functions primarily used	
My overview	1
Status	4
New habits	0
My health data	6
Quiz	3
Would continue app use	
Yes	4
Yes after improvements	5
Maybe	2
No	3

#### Patterns of app use

The majority of users reported frequent app use during the testing period (Table 4). App use was driven by users’ individual contexts, needs, and expectations; the study was not sufficiently powered to to identify patterns of use related to age, educational level, or duration of disease.

The two most frequently used app functions were ‘My health data’, in which users could record health data such as blood sugar, HbA1c, blood pressure, weight, and BMI over time, and ‘Status’, which allowed them to record data related to well-being, sleep, and stress. In contrast, the least frequently used app function was ‘New habits’, which aimed to stimulate reflection about diabetes-related challenges through tests of knowledge and goal setting. Users usually favored one or two functions. Men tended to favor ‘My health data’, whereas the ‘Status’ function was more appealing to women. All users agreed that the function ‘My overview’ was extremely useful for people who were just diagnosed with type 2 diabetes. Several users with diabetes of longer duration stated they would have benefitted from this function in the period immediately after diagnosis.

#### Barriers and facilitators of app use

The development platform was subject to technical issues, such as data entry problems and crashes. According to some users, these technical issues inhibited frequent app use. Two users were ambivalent about continuing to use the app due to these technical issues. The lack of a version for an Android operating system was also mentioned as a barrier. In general, most users had a smartphone or a tablet and were familiar with using apps. They also found the app easy to navigate; only a few users had trouble with navigation. There was no pattern regarding use during test and whether users wished to continue using the app. Three persons stated that they would not continue to use the app. Reasons for this included not experiencing problems with diabetes, general skepticism about technology and lack of time.

#### Barriers and facilitators of implementation

Interviews with users and one focus group with healthcare professionals about implementation of the app revealed two findings of note. The first was related to implementation in practice. Users stated that it was important that the app was introduced by the GP where most patients had received their diagnosis. However, GPs emphasized that the app should also be implemented in other settings, such as community health centers, podiatrists, eye specialists, patients associations, drugstores, etc. Users preferred a brief oral introduction to the app about purpose, content, and download procedures. For users, the introduction by a healthcare professional meant that they trusted the content of the app. Testing showed that the download process and subsequent use were facilitated if users could download the app with a healthcare professional when they preferred to do so.

The other finding was related to technical competencies and knowledge of apps in general. Downloading the app (in particular, the development platform) was challenging for some healthcare professionals. Reasons included a lack of technical competence, lack of knowledge of the app, and lack of experience with apps, iPhone/iPads, or both. Consequently, they found it difficult to introduce and support the app, which caused them to feel less sure about its use. Another reason was the challenge of fitting the app introduction into existing workflow processes due to lack of time, resources, and motivation. Thus, app use was affected by both knowledge among healthcare professionals and their technical competence to support implementation.

## Discussion

People with newly diagnosed type 2 diabetes preferred a multifunctional app to support daily life with diabetes. The prototype app offered five major functions which were informed by users’ needs and ideas: overview of diabetes activities after diagnosis, recording of health data, self-reflection games and goal setting, knowledge games, and recording of psychosocial data, such as sleep, fatigue, and well-being. Users found the app to be a viable tool for support, particularly for increasing their awareness of issues related to sleep, stress, and well-being and for informing and guiding them in the healthcare system after diagnosis. Users during the testing period considered introduction of the app by healthcare professionals as essential to their ability and motivation to download and use the app.

### Multifunctional app vs. a single function app

Simple and understandable design, content, and menu navigation are pivotal and seem to encourage app usability [14]. In addition, recent studies have observed a negative correlation between usability and apps comprising several functions [13]. According to Arnhold et al., the majority of apps offer similar functionalities but combine only one or two of them [14]. The most common functions in diabetes apps involve documentation of data, data forwarding, information function, analysis function, reminder function, but usually only one function per diabetes app [14]. Recorded data often concern psychosocial aspects (well-being), health behaviour (diet and physical activity) or diabetes specific data such as HbA1c, but not the interplay between these factors [13]. With the exeption of data forwarding, our app included all the mentioned functions. However, most users used only one or two functions but favored different functions and different types of recorded data. None of the test users mentioned that the app contained too many functions or that multifunctionaly inhibited their app use. In addition, a clear finding emerged in the design process that users preferred a multifunctional model with the ability to choose between different functions focusing on diabetes specific data and knowledge as well as psychosocial and health behavioural aspects. Similarly, Arnhold et al. argues that multifunctional apps combining documentation, reminder, and advisory functions are more suited for newly diagnosed individuals and elderly people with diabetes [14]. Our study also suggests the value of developing multifunctional apps for individuals newly diagnosed with type 2 diabetes, including the possibility of personalizing apps to individual needs.

Individuals who are newly diagnosed with type 2 diabetes are a diverse group in terms of technical skills, age, individual needs, preferences, diabetes knowledge, and interaction with different healthcare professionals. It is also unknown whether the benefit of the app we developed is confined to only those who are newly diagnosed and how long a diagnosis should be considered new. Some users in our study stated they would not have been ready to use an app during the first years after diagnosis because they had not accepted their diabetes diagnosis. In addition, users who had been diagnosed longer than two years found the function providing an overview of diabetes activities valuable because their diabetes care and prevention activities had changed dramatically since diagnosis. We did not find significant differences in terms of app use or preferences for functions when comparing those diagnosed within the previous 6 months or later. However, including more participants might have enabled analyses of the value of the app to different user groups.

### Implementation of app in the healthcare system

The implementation process for an app is crucial for usability and effect [13]. Most health apps are downloaded by patients through online app stores, and some are introduced to patients as part of their contact with the healthcare system. Some apps are stand-alone solutions with the objective of supporting the patient, and others involve some degree of communication between patients and healthcare professionals or other patients.

In our study, it was evident in the preliminary workshops that a potential app would not be integrated with the separate information technology systems of GPs and community health centers because these systems are not interoperable. In addition, no healthcare professionals were interested in a supplementary system that would operate in parallel to their existing technology, nor did healthcare professionals feel competent or ready to use a mobile app in their consultations with patients. This correlates with other studies finding that lack of human and technical skills are barriers for integration of health technology in practice [8, 10, 22]. A survey among 173 health centers and clinics showed that the three main barriers to implementing cell phone interventions were limited human and technical organizational resources to support implementation, lack of external funding sources to finance investment in mobile technology solutions, and challenges to the technical integration of mobile health solutions with electronic health records and other health information technology infrastructure [22]. These barriers suggest the importance of including healthcare professionals in the development, testing, and implementation processes to create a sense of ownership among healthcare professionals and to identify organizational needs and possibilities.

Users in our study considered it crucial that healthcare professionals introduced the app because it created trust. Some users were afraid of using self-selected apps because they might contain out-of-date or incorrect information. This concern is rational because few apps are research-based, and they may not convey guidelines or content that have been validated by experts [8]. Another issue concerns health economic analysis of health apps. There is a need for studies about apps focusing on both benefits and disadvantages in terms of resources [23].

There are some limitations to this study. Only 14 users tested the app, and results cannot be generalized to all individuals with newly diagnosed type 2 diabetes. Three users stated that they would not continue to use the app. Due to the small number of test users, it is difficult to predict how many newly diagnosed that would in fact accept and use the app in a real life setting. Many users included in the study were well informed about diabetes-related activities, because 10 users were recruited from the community health centers, where recruitment was easier than through GPs. In addition, our criterion for identifying users as newly diagnosed was broad. Consequently, the app requires further testing.

## Conclusions

The co-creation inherent in the design thinking processes during app development and testing were vital to creating value for users. People with newly diagnosed type 2 diabetes found the multifunctional app useful but perceived that introduction to the app by a healthcare professional is crucial for subsequent use. Healthcare professionals may require additional training and guidance to feel comfortable introducing the app to patients.

## Abbreviations

GP: General practice
HbA1c: Glycated hemoglobin
