# Erratum to: development and testing of a mobile application to support diabetes self-management for people with newly diagnosed type 2 diabetes: a design thinking case study

**DOI:** 10.1186/s12911-017-0525-2

**Published:** 2017-09-12

**Authors:** Mira Petersen, Nana F. Hempler

**Affiliations:** Health Promotion Research, Steno Diabetes Center Copenhagen, Copenhagen, Denmark

## Erratum

After publication of the original article [1] it was noted that Figs. 1 and 2 had been swapped.

**Fig. 1:**
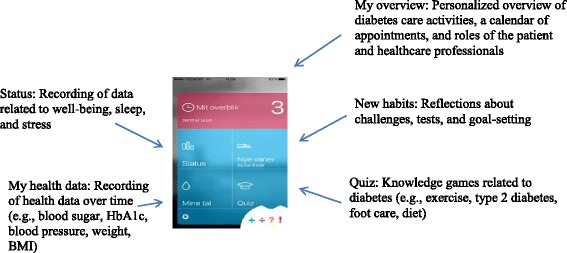
App functions

**Fig. 2:**
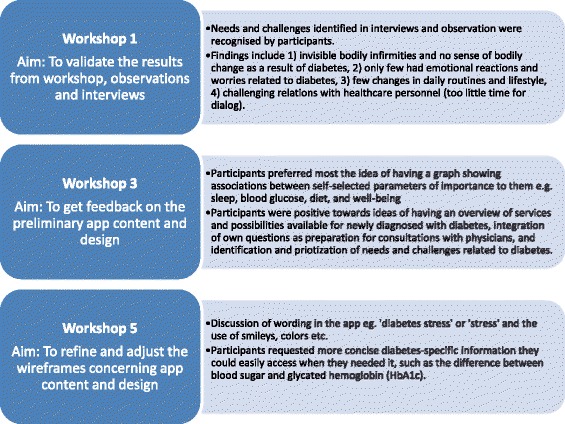
Summary of feedback from workshops with users

Please note that figure one should be swapped with figure two and the corresponding caption. These errors were introduced during typesetting; thus the publisher apologizes for this error. Below are figures one and two with the correct corresponding captions.
